# Spray-drying process preserves the protective capacity of a breast milk-derived *Bifidobacterium lactis* strain on acute and chronic colitis in mice

**DOI:** 10.1038/srep43211

**Published:** 2017-02-24

**Authors:** Patricia Burns, Jeanne Alard, Jiri Hrdỳ, Denise Boutillier, Roxana Páez, Jorge Reinheimer, Bruno Pot, Gabriel Vinderola, Corinne Grangette

**Affiliations:** 1 Univ. Lille, CNRS, Inserm, CHU Lille, Institut Pasteur de Lille, U1019 - UMR 8204 - CIIL - Centre d’Infection et d’Immunité de Lille, F-59000 Lille, France; 2 Instituto de Lactología Industrial (INLAIN, UNL-CONICET), Facultad de Ingeniería Química, Universidad Nacional del Litoral, Santiago del Estero 2829, Santa Fe (3000), Argentina; 3 INTA EEA Rafaela, Ruta 34 km 227, Santa Fe, Argentina

## Abstract

Gut microbiota dysbiosis plays a central role in the development and perpetuation of chronic inflammation in inflammatory bowel disease (IBD) and therefore is key target for interventions with high quality and functional probiotics. The local production of stable probiotic formulations at limited cost is considered an advantage as it reduces transportation cost and time, thereby increasing the effective period at the consumer side. In the present study, we compared the anti-inflammatory capacities of the *Bifidobacterium animalis* subsp. *lactis (B. lactis*) INL1, a probiotic strain isolated in Argentina from human breast milk, with the commercial strain *B. animalis* subsp. *lactis* BB12. The impact of spray-drying, a low-cost alternative of bacterial dehydration, on the functionality of both bifidobacteria was also investigated. We showed for both bacteria that the spray-drying process did not impact on bacterial survival nor on their protective capacities against acute and chronic colitis in mice, opening future perspectives for the use of strain INL1 in populations with IBD.

Inflammatory bowel disease (IBD), comprising Crohn’s disease (CD) and ulcerative colitis (UC), is a chronic relapsing inflammatory disorder. Although the exact pathophysiology of the disease is unknown, the current paradigm is multifactorial and involves a dysregulation of the mucosal immune response towards the gut microbiota in a genetically susceptible host^1^. At present, effective therapeutic management of IBD is lacking: 25–30% of patients fail to respond to current biologics and/or to immunosuppressive drugs, resulting in accumulation of adverse events, including malignancies, and leading to increased levels of opportunistic infections^2^. Therefore new strategies for treating this disease are imperative. While the microbiota is a determinant of normal gut function and immunity, there is increasing evidence that impaired microbiota composition (dysbiosis) plays a crucial role in the onset and maintenance of active IBD^3^,^4^. Therefore targeting this dysbiosis is potentially crucial in the management of the disease.

Probiotics are defined as microorganisms that, when administered in adequate amounts, can confer a health benefit on the host (FAO/WHO^5^), revisited by an expert panel of the International Scientific Association for Probiotics and Prebiotics (ISAPP) in 2013^6^. Probiotics have emerged as a new potential therapeutic strategy in IBD, notably for patients wishing to use “natural and safe” approaches. Following positive results obtained with different animal models of colitis, clinical intervention studies indeed showed beneficial activities of some probiotics in patients suffering from pouchitis or ulcerative colitis^7^. Their protective effects were clearly strain-depended, and several well-characterized probiotic strains failed to fulfil the expected clinical outcome, in particular in patients suffering from Crohn’s disease (CD)^8^. We have previously shown that protection by lactobacilli and bifidobacteria against TNBS (trinitrobenzene sulfonic acid)-induced colitis in mice was strain-specific and correlated well with the *in vitro* immunomodulatory properties of the strains^9^. Strains inducing higher levels of the anti-inflammatory cytokine IL-10 and lower levels of the pro-Th1 cytokines IL-12 and IFNγ after *in vitro* stimulation of human peripheral mononuclear cells (PBMC), offered the best protection in the *in vivo* colitis model used. In contrast, strains inducing a low IL-10/IL-12 cytokine ratio on human PBMC’s could not significantly attenuate colitis symptoms in mice^9^.

Gut microbiota composition depends on dietary habits and lifestyle, and as such the microbiome composition of people from different regions can be different^10^. This offers local source of beneficial microbes with a decrease in the relative costs, thus particularly benefiting subpopulations in the emerging countries^11^. Here we evaluate the anti-inflammatory effects of the strain *Bifidobacterium animalis* subsp. *lactis (B. lactis*) INL1, isolated in Argentina from human breast milk and shown before to display technological^12^ and functional properties in mice^13^,^14^ that make it a potential candidate for probiotic application in IBD.

An increasing demand for probiotics is challenging the industry to produce high amounts of viable cultures in stable forms^15^. Industrial manufacturing processes can significantly alter the structural and functional properties of live microorganisms^16^. While freeze-drying is most commonly used, other drying processes for microorganism preservation have been tested. Spray drying is a low-cost alternative to freeze-drying because it is relatively inexpensive and allows the production of large amounts of dried cells in a continuous process^17^. Particularly in Argentina, many medium to large-size dairy industries possess spray drying infrastructures, which could be exploited for the production of spray dried probiotics. However, spray drying implies somewhat harsher conditions than freeze drying, which might cause membrane damage and cell inactivation. Therefore, the feasibility to apply spray-drying to probiotics will depend on the technological conditions applied and it is anticipated that the impact on the strain functionality might be strain dependent. We thus decided to compare the anti-inflammatory capacities of the Argentinian strain *B. animalis* subsp. *lactis (B. lactis*) INL1 and *B. lactis* BB12, largely described as beneficial in a number of inflammatory conditions^18^ and study the impact of spray-drying on the anti-inflammatory functionality of both strains.

## Results

### The spray-drying did not decrease bacterial survival

Fresh cultures of bididobacteria were spray-dried in 20% (w/v) skim milk (constant inlet air temperature of 137.5 ± 3.5 °C, an outlet temperature of 82.5 ± 7.8 °C and a flux of 600 l/h were used). The mean value of moisture of the powders obtained after spray drying was 3.89% ± 0.09 and 4.22% ± 1.59 (wt/wt) for *B. lactis* INL1 and *B. lactis* BB12, respectively. Both *B. animalis* subsp. *lactis* strains INL1 and BB12 exhibited a satisfactory resistance capacity to spray drying, with a respective survival rate of 97.36 and 98.12% (Table 1). No significant differences in cell counts were observed before and after spray drying.

### The immunomodulation capacities of *B. animalis* subsp. *lactis* INL1 and BB12 strain are similar and not affected by spray-drying

**Figure 1:**
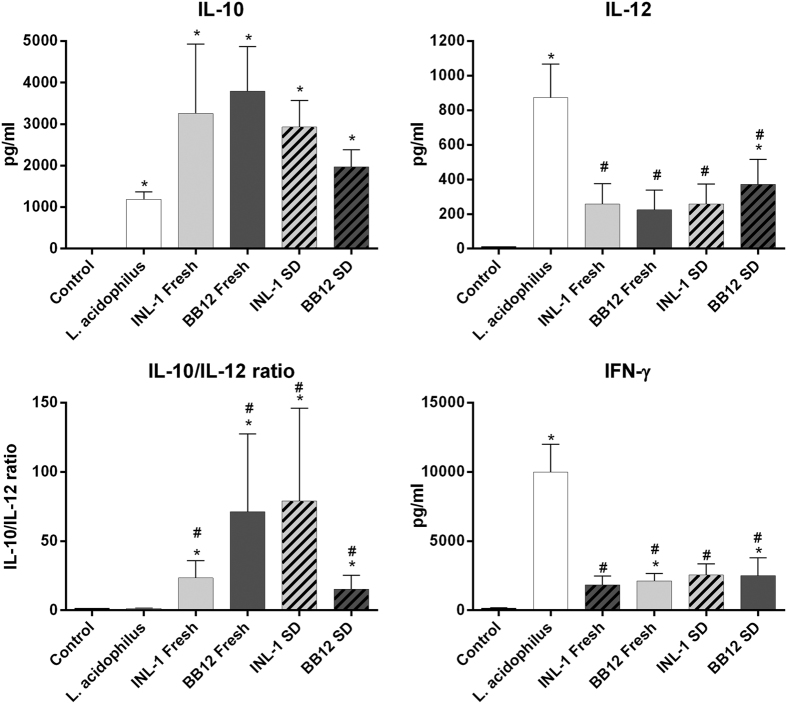
Impact of the drying process on the immunomodulatory potential of *B. lactis* INL1 and BB12. Peripheral blood mononuclear cells (PBMCs, 5 donors per condition) were stimulated *in vitro* at a ratio of 10:1 (bacteria/cells) with fresh or spray-dried *B. lactis* INL1 and BB12 and by fresh *L. acidophilus* strain NCFM (two wells per donor). Results indicated levels of IL-10, IL-12 and IFNγ released by bacteria-stimulated and control human PBMCs for 24 hours and levels of IL-10/IL-12 ratio (mean ± SEM of 5 independent experiments). The indications *or # meaning p < 0.05, refer to the comparison of bifidobacteria-stimulated PBMCs versus untreated cells (*medium control) or versus *L. acidophilus*-stimulated PBMC (# *L. acidophilus* effect), respectively.

The capacity of both bifidobacterial strains to induce the release of cytokines after *in vitro* stimulation of human PBMC’s, was compared (Fig. 1). The cytokine profile observed for INL1 was similar to that of BB12. Indeed both strains were good inducers of the anti-inflammatory cytokine IL-10, while they induced low levels of the pro-Th1 cytokines (IL-12 and IFNγ), leading to a high IL-10/IL-12 ratio in comparison to e.g. *L. acidophilus* NCFM, selected for its strong capacity to induce pro-Th1 cytokines^9^,^19^. Spray drying slightly decreased their capacity to induce IL-10 but both spray-dried bifidobacteria maintained their anti-inflammatory profile as highlighted by the IL-10/IL-12 ratio which remained significantly (p < 0.05) higher than that of the pro-Th1 *L. acidophilus* NCFM strain.

### *B. lactis* INL1 and BB12 strains rescued mice from acute colitis and these protective capacities were not affected by spray-drying

**Figure 2:**
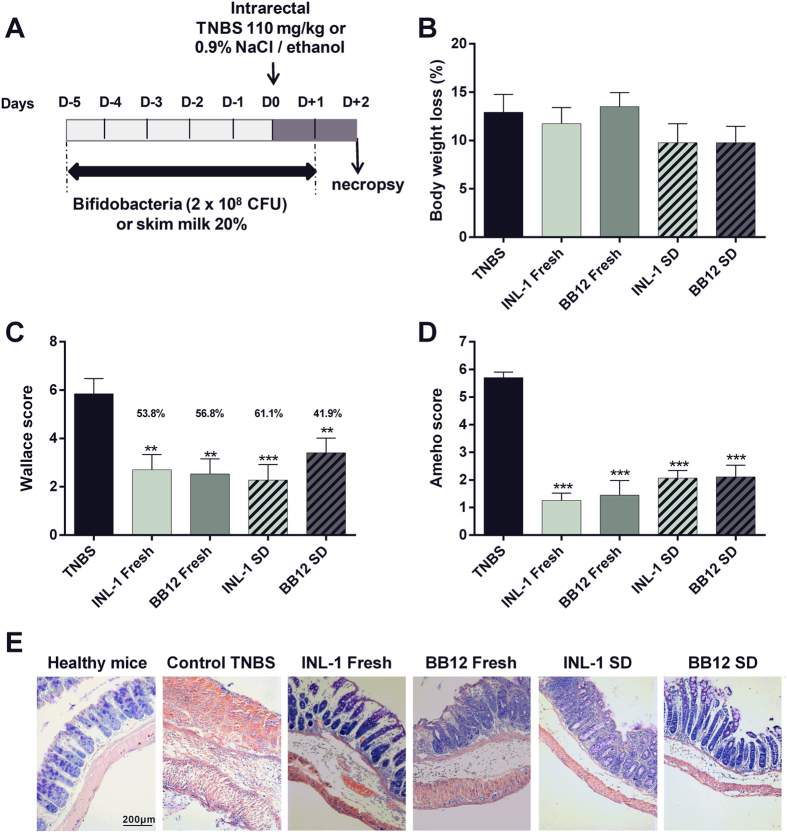
Fresh and spray-dried *B. lactis* INL1 and BB12 alleviated severity of acute TNBS-induced colitis in mice. (**A**) Experimental protocol used for the mouse model of acute colitis induced by intrarectal administration of 110 mg/Kg of TNBS, while control healthy mice received only 50% EtOH. Mice were treated with the probiotics by daily intragastric administration (2 × 10^8^ CFU in 200 μl 20% (w/v) skim milk) or by 20% (w/v) skim milk (control healthy mice and control TNBS) for five consecutive days before colitis induction (intrarectal administration of TNBS at 110 mg/Kg) and 1 day after TNBS administration. (**B**) Body weight loss (as a percentage of the initial weight at the day of colitis induction). (**C**) Macroscopic evaluation of colonic inflammation (Wallace score). Percentages of protection are indicated above each bar (**D**) Histologic evaluation of colonic inflammation (Ameho score). (**E**) Representative histological sections (stained by May Grünwald Giemsa, 100X magnification) of mice treated with skim milk and administrated (Control TNBS) or not (heathy mice) with TNBS, and mice that were treated with fresh or spray-dried *B. lactis* INL1 and BB12 before and one day after TNBS administration. The data represent the mean ± SEM of 10 mice per group of a representative experiment. Two different experiments were performed. **p < 0.01 or ***p < 0.001 versus the TNBS control group. For histology, 5 sections of each mouse (n = 10 per experimental group) were analyzed.

**Figure 3:**
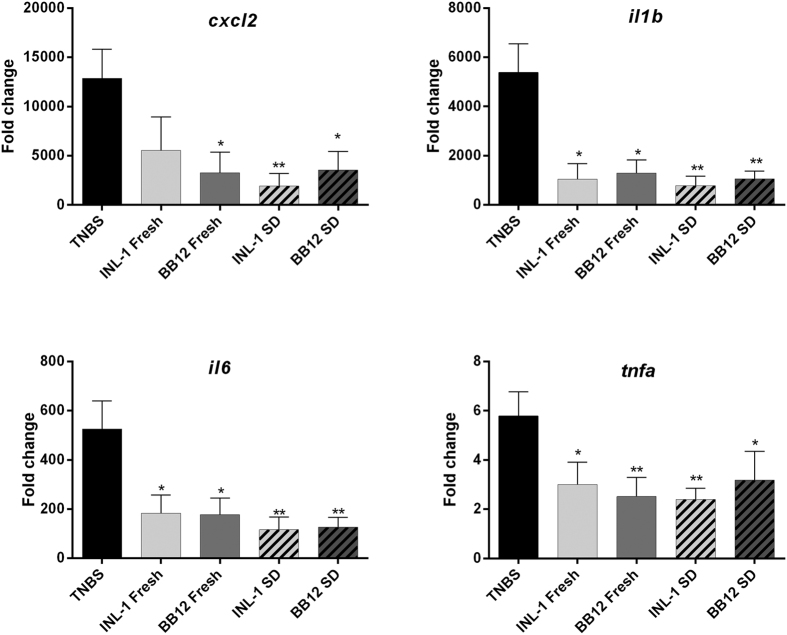
Fresh or spray-dried *B. lactis* INL1 and BB12 dampened colonic inflammatory gene expression in the acute TNBS-induced colitis model. Gene expression of *Cxcl2, il1b, tnfa, il6* in colonic samples harvested two days after colitis induction was assessed by real time qPCR. Values are expressed as the relative mRNA levels compared with colons from healthy mice. Data represent mean values of 10 animals ± SEM. *p < 0.05; **p < 0.01, in comparison to TNBS control samples.

To compare the *in vivo* anti-inflammatory capacities of both INL1 and BB12 strains, we first used a well-established murine model of acute colitis (Fig. 2A), induced by TNBS (110 mg/Kg)^9^,^20^. As expected, TNBS treatment (after 48 h induction) reduced body weight (by 13%; Fig. 2B) and induced a strong colitis, highlighted both by a high macroscopic score (Wallace score of 5.9 ± 0.6, Fig. 2C) as well as a high histological score (Ameho score of 5.7 ± 0.2, Fig. 2D,E). Both fresh cultures of *B. lactis* INL1 and BB12 were able to significantly protect mice from colitis, as shown by a strong and significant reduction of both macroscopic (p < 0.01, Fig. 2C) and histological (p < 0.001, Fig. 2D,E) scores of inflammation, leading to a protective effect of 53.8% and 56.8%, respectively. This protective effect was not significantly affected by spray drying since both spray-dried strains were also able to reduce the macroscopic (p < 0.001 and 0.01 for INL-1 and BB12, respectively) and histological (p < 0.001) scores of inflammation, reaching 61.1% protection for INL1 (Fig. 2). The anti-inflammatory capacity of both strains, whether fresh or spray-dried, was confirmed by following the colonic mRNA expression of pro-inflammatory cytokines and chemokines, showing a strong and significant (p < 0.05 and 0.01) downregulation of the gene expression of all the inflammatory markers tested (CXCL2, IL-1β, TNF-α, IL-6) in comparison to control TNBS-treated mice. The latter, because of the TNBS treatment exhibited a strong upregulation of these pro-inflammatory genes (Fig. 3) in comparison to control healthy mice that received only skim milk but not challenged with TNBS (normalized to 1).

### The anti-inflammatory capacity of fresh and spray-dried forms of *B. lactis* INL1 and BB12 strains was confirmed in a model of chronic colitis

**Figure 4:**
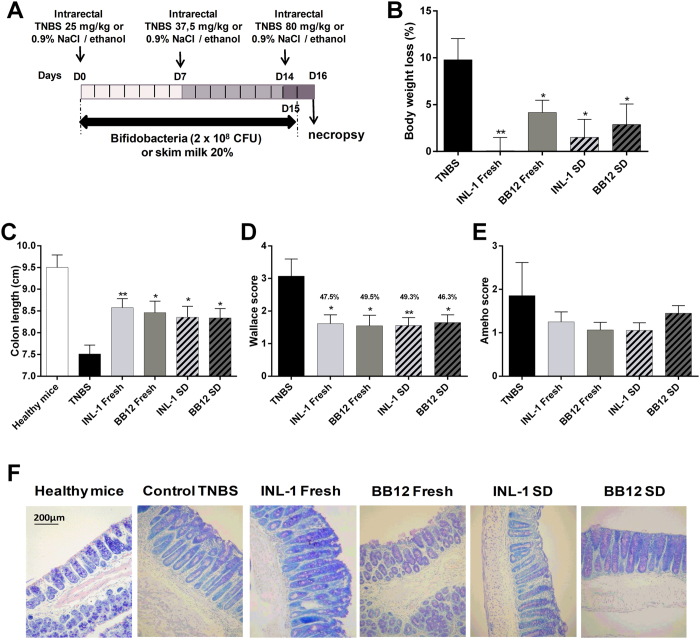
Fresh and spray-dried *B. lactis* INL1 and BB12 rescued mice from chronic TNBS-induced colitis. (**A**) Experimental protocol used for the mouse model of chronic colitis induced by intrarectal administrations of 3 increasing doses of TNBS (25 mg/kg, day 0; 37.5 mg/kg, day 7 and 80 mg/kg, day 14), at one week interval, while control healthy mice received only 50% EtOH. Mice were treated with the probiotics by daily intragastric administration [2 × 10^8^ CFU in 200 μl 20% (w/v) skim milk) or by 20% (w/v) skim milk (control healthy mice and control TNBS)] every day during colitis induction (from D0 to D15). (**B**) Body weight loss (as a percentage of the initial weight). (**C**) Colon length at sacrifice (Day 16). (**D**) Macroscopic evaluation of colonic inflammation (Wallace score). Percentages of protection are indicated above each bar (**E**) Histologic evaluation of colonic inflammation (Ameho score). (**F**) Representative histological sections (stained by May-Grünwald-Giemsa, 100X magnification) of mice treated with skim milk and administrated (Control TNBS) or not (heathy mice) with TNBS, and mice that were treated with fresh or spray-dried *B. lactis* INL1 and BB12 during all the experiment. The data represent the mean ± SEM of 10 mice per group of a representative experiment. Two different experiments were performed. *p < 0.05, **p < 0.01 versus the TNBS control group.

**Figure 5:**
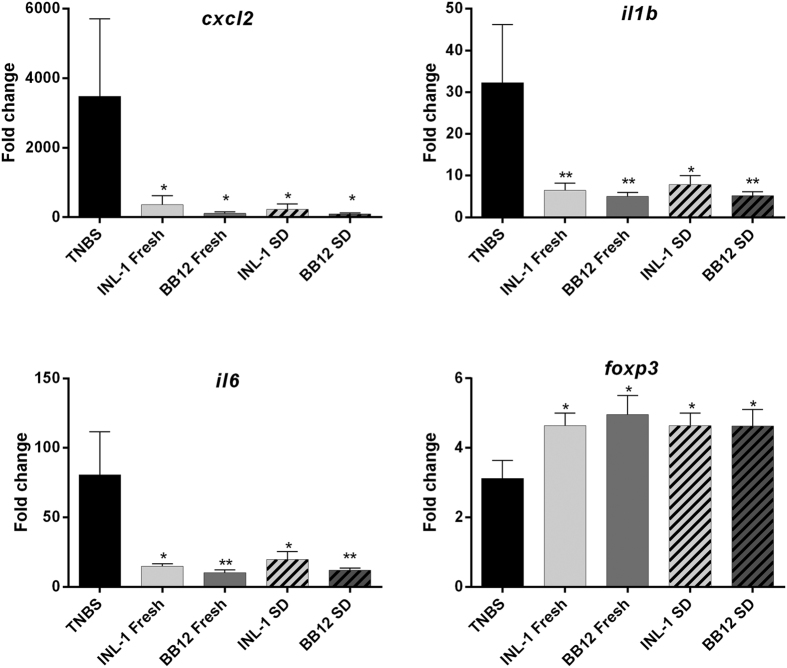
Fresh or spray-dried *B. lactis* INL1 and BB12 abrogated colonic inflammatory gene expression and increased *foxp3* in the chronic TNBS-induced colitis model. Gene expression of *Cxcl2, il1b, il6 and foxp3* in colonic samples harvested two days after the last TNBS administration (D16) was assessed by real time qPCR. Values are expressed as the relative mRNA levels compared with colons from healthy mice. Data represent mean values of 10 animals ± SEM. *p < 0.05; **p < 0.01, in comparison to TNBS control samples.

The model of chronic TNBS inflammation used in this study involves the induction of a low grade, subclinical inflammatory status, mimicking the chronic nature of inflammation in IBD^21^. Chronic low-grade inflammation was induced in mice by the administration of 3 subclinical, but increasing doses of TNBS (Fig. 4A). This led to a moderate body weight loss (9.8% ± 2.3, Fig. 4B), decrease of colon length (7.5 ± 0.2 cm versus 9.5 ± 0.5 cm for healthy control mice, Fig. 4C), and the induction of a mild macroscopic (Wallace score of 3.1 ± 0.5; Fig. 4D) and histological (1.9 ± 0.8; Fig. 4E,F) inflammation scores, as compared to the acute colitis model. The chronic colitis model also led to a significant upregulation of inflammatory gene expressions (Fig. 5) that were, however, less severe than those observed for acute colitis (Fig. 3). As for acute colitis, both *B. lactis* strains were able to counteract the inflammation status, showing significant (p < 0.05 or 0.01) decrease in weight loss and macroscopic score of inflammation, significant increase (p < 0.05 or 0.01) of colon length and a slight decrease of the histological score of inflammation (Fig. 5), in comparison to TNBS-treated control animals. Both strains were also able to significantly (p < 0.05 and 0.01) decrease, with the same efficiency, the expression of pro-inflammatory genes (*cxcl2, Il1b and il6*). In this model we also checked the capacity of the probiotics to induce the expression of *foxp3* which is a marker of regulatory T cells. We indeed observed a significant (p < 0.05) increase of the expression of this gene in all probiotic-treated animals in comparison to TNBS control mice (Fig. 5). Again, spray-dried and fresh bacteria displayed the same efficacy in counteracting chronic colitis.

## Discussion

The increased understanding of the interactions between the gut microbiota and the host immune system has raised the interest towards a targeted modulation of intestinal bacterial communities as a novel potential therapy in IBD. Similar to the findings in animals, several studies showed a significant alteration of the microbiota in IBD patients^22^. Significantly lower counts of *Faecalibacterium prausnitzii* but also of bifidobacteria were found in the rectal biopsies of patients with UC, as compared to healthy patients^23^,^24^. While promising outcomes have been observed after gut microbiota modulation through probiotic supplementations in UC patients^25^,^26^,^27^, disappointing results were obtained for CD patients (see systematic review^7^). It is noteworthy that the effects are considered strain-specific and more studies are needed to understand the underlying mechanisms of action prior to their prophylactic or therapeutic use in IBD patients^7^,^28^.

The selection of probiotic strains should therefore be driven by the needs identified at the level of the host (infection, inflammation, barrier deficiency) and the *in vitro* and *in vivo* established immunological, microbiological and/or metabolic effects of the microorganisms to be considered. In the above philosophy, it is very important that further research is conducted in order to better understand the interactions between the gut microbiota and the host, in states of health as well as in states of dysbiosis. Bifidobacteria are normal residents of the human intestine, commonly found in the infant gut and particularly enriched in the intestine of breastfed infants^29^. Their role early in life is to promote a tolerogenic immune system in the neonate, in order to reverse the Th2 skewed immune system inherited from the pregnant mother^30^,^31^. The latter is promoted by several mechanisms, including the promotion and delivery of bifidobacteria to the newborn via breast milk^32^. Breast milk has shown to be a continuous source of commensal, potentially probiotic, bacteria for the infant gut. Bifidobacteria in particular are known to play a crucial role in the prevention of a variety of immune-mediated diseases such as allergy and IBD^29^,^33^.

In the present study, we thus evaluated *B. animalis* subsp. *lactis* INL1, a strain isolated from human breast milk as a potential candidate for probiotic application in patients suffering from IBD. We notably compared its anti-inflammatory capacities to the well-known probiotic strain *B. lactis* BB12, which is largely recognized for its probiotic properties including its anti-inflammatory capacity^34^. Moreover, we evaluated in both strains the impact of spray-drying process on their anti-inflammatory functionality. Spray-drying is an economically interesting process for the preparation of industrial quantities of viable microorganisms, the cost of which is only 12–20% of the cost of freeze drying^35^. While currently mostly used to prepare food additives, it recently received considerable interest for the production of probiotics. Preliminary experience, however, showed that the successful application of the process is strain-dependent^17^,^36^, mainly because of the high processing temperatures used^17^. High temperatures may potentially reduce strain viability and could lead to important variations in bacterial hydrophobicity and immunomodulation properties.

We first showed that for both strains investigated, the spray drying process in a 20% (w/v) skim milk carrier, had no deleterious impact on bacterial survival, confirming earlier observations for *Bifidobacterium* species, which were shown to have a high resistance to spray drying^37^. Moreover, we found that both *B. lactis* strains exhibited a similar *in vitro* anti-inflammatory cytokine induction profile (high IL-10 versus IL-12 ratio), and that this immunomodulation capacity was not affected by the spray-drying process. Laconelli *et al*.^38^ recently reported that the effects on cell functionality of dehydration treatment are not directly linked to cell survival, and they illustrated that each bacterial strain had a specific sensitivity to the three different drying processes used. Spray-drying performed extremely well in terms of viability and functionality, mainly because it decreased the *in vitro* induction of pro-inflammatory/proTh1 cytokines. In the present study with bifidobacteria, spray-drying did not significantly impact their capacity to induce IL-10 nor the pro-Th1 cytokines IL-12 and IFNγ.

The anti-inflammatory potential of both *B. lactis* strains was further confirmed using a standardized acute TNBS-induced colitis model^9^,^20^. Both *B. lactis* strains were able to rescue mice against acute colitis and were also able to limit the inflammation levels as confirmed by the strong decrease of gene expression for pro-inflammatory cytokines.

We also set up a model of chronic colitis, induced by the administration of 3 subclinical but increasing doses of TNBS. We observed an induction of low grade, subclinical inflammation, with mild macro- or microscopic damages and lower pro-inflammatory cytokine induction, as compared to the acute TNBS model. Therefore the chronic model is thought to better mimic the chronic nature of inflammation in IBD patients. Both *B. lactis* strains were again able to rescue mice from chronic colitis, with similar levels of protection as observed in the acute colitis model, and were also able to limit inflammation levels, as measured by cytokine gene expression. Regulatory T (Treg) cells are known to play a critical role in the control of inflammatory response. Transcriptional factor FoxP3 serves as a lineage specification factor of these cells^39^. In the chronic model of colitis, we showed that both *B. lactis* strains increased the expression of *foxp3,* suggesting that a desired T-regulatory cell response could be involved in the protective effect.

Importantly, the bacterial functionalities of both strains were not affected by spray-drying. Spray-dried bacteria were able to decrease the macroscopic and histologic scores of inflammation, and dampened the pro-inflammatory cytokine expression. Spray-dried bifidobacteria were also able to upregulate *foxp3* gene expression at the same level as fresh cultures, indicating that spray-drying did not alleviate their immune-regulatory capacities. This confirms previous studies performed with probiotic lactobacilli which showed that spray-drying did not impact e.g. the induction of immunoglobulin A (IgA)-producing cells in mice^36^. Therefore spray-drying appears to be an interesting process for the stabilization of probiotic bifidobacteria.

In conclusion, these results provide further evidence of the probiotic potential of the *B. lactis* INL1 strain, and particularly for an application in the attenuation of gut inflammation. Moreover, since bacterial functionalities were not affected by spray-drying, this preservation process appears to be an interesting process for the stabilization of this strain. Based on our data, it can be concluded that the strain INL1 has the potential to be explored as prospective functional therapy to manage IBD. Further studies should be conducted to investigate the mechanism of action of the bacterium and to establish its clinical efficacy in well-designed double-blind placebo-controlled human trials.

## Methods

### Microorganisms and growth conditions

*B. animalis* subsp. *lactis* INL1, whose functional and technological properties were previously reported^12^,^13^,^14^, was used in this study and compared to the commercial strain *B. animalis* subsp. *lactis* BB12. *L. acidophilus* NCFM kindly provided by DuPont^TM^ Danisco (Madison, WI, USA). was also grown overnight at 37 °C in de Man, Rogosa and Sharpe broth (MRS, Difco, Detroit, USA) and used as pro-Th1 control strain^9^. Bifidobacteria were cultured overnight at 37 °C in anaerobic condition (GENbag anaer, Biomérieux, France), in MRS supplemented with 0.1% (w/v) L-cysteine hydrochloride (Sigma), washed twice in sterile PBS and resuspended in PBS for *in vitro* studies and in 20% (w/v) skim milk for *in vivo* experiments. Spray-dried cultures were resuspended in distilled water to a 20% (w/v) of total solids, in order to have fresh and spray-dried cells resuspended in the same medium.

### Spray drying in skim milk

Overnight cultures of both bifidobacteria strains in MRS broth with 0.1% (w/v) L-cysteine hydrochloride were harvested (6000 × *g*, 15 min, 5 °C), washed twice with PBS and re-suspended in 20% (w/v) skim milk. Cell suspensions were spray-dried on a laboratory scale spray-dryer (Buchi mini spray dryer model B290, Flawil, Switzerland). A constant inlet air temperature of 137.5 ± 3.5 °C, an outlet temperature of 82.5 ± 7.8 °C and a flux of 600 l/h were used. Cell suspensions were atomized and sprayed into the drying chamber by using a two-fluid nozzle. The product dried almost instantaneously and the residence time was negligible. Three independent replicates were performed for each strain. Spray dried powders were vacuum sealed in individual samples of 10 g. Residual moisture (% wt/wt) was determined in triplicate at 101 ± 1 °C (FIL-IDF 26 A: 1993^40^). Cell counts of bifidobacteria were performed before and after spray drying on MRS with 0.1% (w/v) L-cysteine hydrochloride agar (37 °C, 48 h anaerobic incubation).

### Bacterial enumeration

Bifidobacteria suspensions were serially diluted in PBS buffer, plated on MRS agar supplemented with 0.1% (w/v) L-cysteine hydrochloride and grown under anaerobic condition (GENbag anaer, Biomérieux, France) at 37 °C. Colonies were counted after 48 h of incubation.

#### *In vitro* immunomodulation assays

Blood samples were obtained from five healthy informed donors. All experimental protocols were approved by our institution committees (INSERM, CNRS and Institut Pasteur de Lille) in accordance with relevant guidelines and regulations. Blood sampling from healthy informed donors were done upon approved agreement of volunteers (signed consents) by authorized staff in accordance with the abovementioned committees, i.e. (INSERM, CNRS and Institut Pasteur de Lille). Peripheral blood mononuclear cells (PBMCs) were isolated from the blood as described before^9^. Briefly, after Ficoll gradient centrifugation (Pharmacia, Stockholm, Sweden), mononuclear cells were collected, washed in RPMI-1640 medium (Life Technologies, Ghent, Belgium), and adjusted to 2 × 10^6^ cells per ml in RPMI supplemented with gentamicin (150 μg/ml), glutamine (2 mM), and 10% fetal calf serum (Gibco). PBMCs (two wells per donors) were stimulated with phosphate-buffered saline or bacteria (fresh cultured or spray dried bacteria at a bacterium-to-cell ratio of 10:1) for 24 h at 37 °C with 5% CO_2_. The supernatants were collected and stored at −20 °C until cytokine measurements performed using R&D Duoset ELISA kits (R&D, Minneapolis, MN, USA). ELISA was performed in duplicate for each sample.

### Experimental TNBS-induced colitis models and study design

Female BALB/c ByJ mice (7–8 weeks old) were purchased from Charles River Laboratories (L’Arbresle, France) and were maintained under specific pathogen-free conditions. Animal experiments were performed in an accredited establishment (number A59107; animal facility of the Institut Pasteur de Lille, France) and carried out in accordance with the guidelines of laboratory animal care published by the French Ethical Committee and the rules of the European Union Normative (number 86/609/EEC). All the studies were approved by the local investigational ethics review board (Nord-Pas-de-Calais CEEA N°75, Lille, France; protocol reference numbers 192009 R). The animal experiments also complied with French legislation (Government Act 87–848) and the European Communities Amendment of Cruelty to Animals Act 1976. Before experimentation, animals were provided a one week acclimation period and were given *ad libitum* access to regular mouse chow and water.

A standardized murine TNBS colitis model was used to induce acute levels of inflammation in mice (Fig. 2A). Briefly, mice (n = 10 per each experimental group) were randomly co-housed in two cages per group (n = 5/cage) and the experiment started after one week acclimation. The anti-inflammatory properties of the bifidobacteria were evaluated by the intra-gastric administration of live bacteria (2 × 10^8^ CFU of freshly cultured or spray-dried bacteria in 20% (w/v) skim milk) or 20% (w/v) skim milk alone (for the control healthy or TNBS-treated mice) once daily, for five consecutive days before colitis induction and 1 day after the TNBS administration. For acute colitis induction, mice were anesthetized and received an intrarectal administration of a 50 μl solution of the hapten reagent TNBS (Sigma-Aldrich, France) dissolved in 0.9% NaCl/ethanol (50/50 v/v) at a final concentration of 110 mg/Kg, while control healthy mice received only 50% EtOH.

The chronic model of colitis was performed by administration of 3 increasing doses of TNBS with one week interval as described in Fig. 4A (n = 10 mice per each experimental group randomly co-housed in two cages per group; n = 5/ cage). Briefly, anesthetized mice received intrarectally 50 μl solution of TNBS (Sigma-Aldrich, France) at a dose of 25 mg/Kg (day 0), 37.5 mg/Kg (day 7) and 80 mg/Kg (day 14), dissolved in 0.9% NaCl/ethanol (50/50 v/v), while control healthy mice received only 50% EtOH. The anti-inflammatory properties of the bifidobacteria were evaluated by the daily intra-gastric administration of live bacteria (2 × 10^8^ CFU of freshly cultured or spray-dried bacteria in 20% (w/v) skim milk) or 20% (w/v) skim milk (for the TNBS control or healthy control mice) every day during all the experimental procedure (from D0 to D15).

Colons were removed at sacrifice (48 h after the last TNBS administration), washed and opened. Inflammation grading was performed blindly using the Wallace scoring method, reflecting both the intensity of inflammation and the extent of the lesions^41^. Protection was calculated using the following formula: 100 x [(mean score of TNBS group – mean score of experimental group)/mean score of TNBS group]. Histological analysis was carried out on May-Grünwald Giemsa stained 5 μm tissue sections from colon samples (5 sections per mice) fixed in 4% formalin, embedded in paraffin and blindly scored according to the Ameho scoring method^42^. For RT-PCR analysis, a part of the colon corresponding to the inflamed region was cleaned from fecal material and stored in RNA-later^®^ buffer (Ambion, Life Technologies, Austin, TX, USA) at –80 °C until RNA extraction.

### RNA extraction and qRT-PCR

Tissue samples were homogenized using Lysing Matrix D (MPbio). Total RNA was extracted using Macherey-Nagel NucleoSpin RNAII isolation kit according to the manufacturer’s recommendation. Quantity and quality of RNA were checked by nanodrop (260/280 nm, 260/230 nm). 260/280 ratio was higher than 1.95 in all samples. RNA (1 μg) was reverse-transcribed using the High capacity cDNA reverse transcription kit (Applied Biosystems^TM^, Foster City, CA, USA). Real-time quantitative PCR (RT-qPCR) was performed using the Power SYBR Green PCR Master Mix (Applied Biosystems) on the ABI-ViiA7 Real Time PCR system. The forward and reverse primers used in the study were, respectively as follows: *b-actine*: 5′- CTAAGGCCAACCGTGAAAAC -3′ and 5′- ACCAGAGGCATACAGGGACA-3′; il1b: 5′-TTGACGGACCCCAAAAGATG-3′ and 5′-AGAAGGTGCTCATGTCCTCA-3′; tnfa: 5′-CCCTCACACTCAGATCATCTTCTC-3′, and 5′-GGCTACAGGCTTGTCACTCG-3′; cxcl2: 5′-CAAAAGATACTGAACAAAGGCAA-3′ and 5′-TCAGGTACGATCCAGGCTTCC-3′; il6: 5′-AGCCAGAGTCCTTCAGAGAGATAC-3′ and 5′-AATTGGATGGTCTTGGTCCTTAGC-3′; foxp3: 5-TCCTTCCCAGAGTTCTTCCA-3′ and 5′-AAGTAGGCGAACATGCGAGT-3′. The 2^−Δ(ΔCT)^ method was used to normalize gene expression levels. Relative mRNA levels (2^−Δ(ΔCT)^) were determined by comparing 1) the PCR cycle thresholds (Ct) for the gene of interest and the housekeeping gene *Actb* (ΔCt) and 2) ΔCt values for treated and healthy control animal groups (ΔΔCt).

### Statistics

GraphPad Prism was employed for graph preparation and statistical evaluation. Differences between groups were assessed using ANOVA, followed by nonparametric Mann-Whitney test. Data with p value ≤ 0.05 were considered to be significant.

## Additional Information

**How to cite this article**: Burns, P. *et al*. Spray-drying process preserves the protective capacity of a breast milk-derived *Bifidobacterium lactis* strain on acute and chronic colitis in mice. *Sci. Rep.* **7**, 43211; doi: 10.1038/srep43211 (2017).

**Publisher's note:** Springer Nature remains neutral with regard to jurisdictional claims in published maps and institutional affiliations.

## Figures and Tables

**Table 1:** Resistance of *B. lactis* INL-1 and *B. lactis* BB12 to spray drying in 20% (w/v) skim milk.

Strains	Cell count (log CFU/ml)		Survival rate (%)
	Before SD	After SD	
*B. lactis* INL-1	10.19 ± 0.08	9.92 ± 0.08	97.36 ± 1.44
*B. lactis* Bb12	10.10 ± 0.07	9.91 ± 0.10	98.12 ± 0.94

Mean ± SD. No significant differences in cell counts before and after spray drying, according to paired t-test (p > 0.05).
